# Comparative study for removal of phosphorus from aqueous solution by natural and activated bentonite

**DOI:** 10.1038/s41598-022-23178-w

**Published:** 2022-11-12

**Authors:** Abdelbaky Hossam Elgarhy, Belal N. A. Mahran, Guanglong Liu, Talaat A. Salem, ElSayed ElBastamy ElSayed, Lubna A. Ibrahim

**Affiliations:** 1 Central Laboratory for Environmental Quality Monitoring (CLEQM), NWRC, Qalyobia, 13621 Egypt; 2 State Environmental Protection Key Laboratory of Soil Health and Green Remediation, College of Resources and Environment, Huazhong Agricultural University, Wuhan, 430070 China; 3 Nile Research Institute, National Water Research Center (NWRC), Qalyobia, 13621 Egypt; 4 Water Management Research Institute (WMRI), National Water Research Center (NWRC), Qalyobia, 13621 Egypt

**Keywords:** Environmental sciences, Environmental chemistry

## Abstract

The novelty of the current article is to investigate the adsorption potential of the Egyptian natural and activated bentonite (Na-bentonite) to inorganic and organic phosphorus (IP, OP) in aqueous media. The natural bentonite (NB) was activated to Na-bentonite (Na-B) by a new facile method within 2 h. NB and Na-B were also characterized using XRD, XRF, BET ESM, and FT-IR. The batch experiment has been employed to select the ideal conditions for the removal of inorganic and organic phosphorus (IP, OP) from aqueous solutions. The findings clearly showed that the Na-bentonite is enriched with sodium in the form of Na-montmorillonite with a higher specific area 138.51 m^2^/g than the value for the natural bentonite 74.21 m^2^/g. The batch experiment showed maximum absorption for both IP and OP adsorbents occurred at an equilibrium pH = 6, contact time of 40 to 50 min, 40 °C temperature, and a dose rate of 2 mg/L and 1 mg/L, respectively. The equilibrium data displayed better adjustment to Langmuir than the Freundlich, Temkin, and Dubinin-Radushkevich isotherms and adsorption kinetics followed the pseudo-second-order-type kinetic, and the parameters of thermodynamics reveal that adsorption occurs spontaneously and exothermic nature. Na-bentonite proved to be more efficient in removing target material than natural bentonite. The spent bentonites were easily regenerated by chemical methods.

## Introduction

Phosphorous (P) is a fundamental element for plant and animal growth, and its use has long been recognized as critical to crop and livestock production profitability. Therefore, it meets the worldwide food prerequisites^1^. However, the transfer of phosphorus (P) from soils to water bodies has recently been a public concern^1^. Where increased P discharge into natural water systems contributed significantly to eutrophication. Since the late 1960s, the wastewater’s phosphorus abatement has obtained a lot of attention. P has been removed from water using a variety of processes, including reverse osmosis, biological denitrification, electro-dialysis, and adsorption. Most of these strategies proved to be better suited to control high P concentrations^2^. Low P concentrations were notoriously difficult to manage, so adsorption was recommended as one of the most effective removal techniques^3^. The most important qualities of the adsorption method that demonstrated its high efficiency were quick handling, easy access to diverse adsorbents, and low-cost materials^4^.

The selection of an adequate adsorbent is critical to the performance of an adsorption technique^5^. The first two pathways, ligand exchange (adsorption), precipitation, lattice diffusion, and anion exchange, are the most essential for phosphorus sorption^6^. Adsorption and ion-exchange characteristics are seen in many natural materials. Most of the crystals are aluminosilicates with cation exchange properties that form natural ion exchange materials. However, some aluminosilicates, like zeolites^7,8^, bentonite^8,9^, and diatomite^8,10^, can operate as anion exchangers. Synthetic and natural adsorbents, or a combination of both, are used as efficient solid materials for adsorption. In recent years, there were a lot of research into removing phosphors from solutions using organic wastes and solid wastes. Synthetic adsorbent materials have a better adsorption capability than clay minerals due to their homogeneous micropore structure and large surface area^11^.

Egypt has some significant clay mineral resources. The Egyptian Mineral Resources Authority (EMRA) estimated the country’s major mineral resources to include 5 billion metric tons (BMT) of silica sand, 1.25 BMT of phosphate, 1 BMT of feldspar, 0.9 BMT of iron ore, 0.224 BMT of nonferrous metals, and 0.15 BMT of bentonite^12^.

In the last years, researchers have investigated a variety of solid adsorbents to validate if they can be used to reduce phosphorous levels in waterways. Natural Clay minerals such as bentonite, kaolinite, and zeolite and their modified forms, carbonates, and fly ash, have initiated a renewed interest in the removal of many pollutants such as phosphors, cations, and anions by ion exchange, adsorption, or both from aqueous solutions and wastewater due to their low cost and eco-friendly^8,13–19^.

Bentonites possess a special position among clay raw materials because of the unique properties of smectites, which consist mainly of calcium and/or sodium montmorillonite^8,20^. These days soda activation of bentonite rich in Ca-smectite is a standard industrial practice in manufacturing drilling muds, adsorbents, barrier clays, binders, etc. Transformation to Na-form is also commonly employed in multi-stage modifications utilized in the engineering of advanced clay-based materials, as the first step facilitating subsequent phases of the process. Due to its significance and widespread use, the Na-activation of bentonites was in the scope of scientists’ interest which led to publish several papers addressing this issue^20–24^. The procedures of activation outlined in the literature^20–24^ include the preparation of mixtures of bentonite, and sodium carbonate (Na_2_CO_3_), at different water proportions, temperatures, and times of interactions (more than two h). Hence, in this study novel procedure for the preparation of Na-bentonite (Na-B) within two hours was investigated.

The main aim of the current research is to find safe, low-cost, effective, and available materials in the Egyptian environment that can reduce the pollution load from different waterways in a safe and easy way without any negative impact on the environment. The objective of this investigation is to prepare Na-bentonite by a newer facile method than the current one, regarding the literature, and applied in the laboratory under various operating conditions to adsorb IP and OP. The adsorption experiments were performed under various conditions. Four isotherm models (Langmuir, Freundlich, Temkin, and Dubinin-Radushkevich) and two kinetics (pseudo-first-order and pseudo-second-order), and the thermodynamic studies were used to analyze aquarium data. Regeneration of spent materials and cost was estimated.

## Material and methods

### Chemical and reagents

All reagents and chemicals used in this research were analytical grade. Double distilled—deionized water was used for the preparation of adsorbents and phosphorus solutions. Potassium dihydrogen phosphate (99% pure) was purchased from Merck, while sodium carbonate was from Sigma.

### Preparation of natural and activated bentonite (Na-bentonite)

The bentonite sample used in this research was obtained from Masr Company for Mineralization and Bentonite, Burg El-Arab, Egypt. The sample was first crushed using a hammer on a hard surface, then ball-milled, and only particles smaller than 0.10 mm, were used for the batch experiments. To remove non-adhesive impurities and particles, the sample of natural bentonite was washed with deionized water, and then, dried at 70 °C for 24 h to get rid of the moisture^25^.

A 200 g sample of Ca-bentonite was oven-dried for 2 h at 300 °C to eliminate the interlayer water from montmorillonite. After having the dried sample from the oven removed, it was directly subjected to impregnation in a solution of 22.2% (wt/vol [water]) sodium carbonate (Na_2_CO_3_) [ratio of Ca-bentonite/Na_2_CO_3_ = 9/1]. The maximum exchange was achieved by boiling with vigorous stirring the mixture for 60 min, after that the solution was cooled and let to settle down. The settled clay was washed several times with deionized water and dried in the oven, and finally left in a desiccator until use in the experiments^20,26^. The exchange between sodium carbonate and Ca-bentonite can be illustrated in Eq. (1).

1 $${\text{Ca}}^{2 + } - {\text{Bentonite }} + {\text{ Na}}_{2} {\text{CO}}_{3} \rightleftharpoons 2{\text{ Na}}^{ + } - {\text{Bentonite }} + {\text{ CaCO}}_{3} \downarrow$$

### Characterizations of natural and activated bentonite (Na-bentonite)

The mineralogical properties of natural and Na-bentonite were determined by X-ray Powder Diffraction (XRD) using a PAN analytical X-ray Diffraction equipment model X Pert PRO, supported with CuKα radiation under target voltage 40 kV and current 30 mA in a scanning rate of 2° (2θ)/min. The chemical composition of each adsorbent was analyzed using X-ray Fluorescence (XRF) spectroscopy (Axios, sequential WD- XRF spectrometer, PAN analytical^27^.

The surface area is an important factor in determining the active sites on adsorbents that will become occupied by the IP and OP. Nitrogen sorption experiments were performed using a TriStar 3000 V6.05 A System. The surface area of the two adsorbents was investigated by applying the Brunauer-Emmet-Teller (BET) method^28^.

The investigation of FTIR for both adsorbents allowed spectrophotometric observation and provide means to identify the functional groups on the adsorbent surface. FTIR Spectroscopy, Attenuated Total Reflection (FTIR-ATR) was performed to clarify the differences between natural and Na-bentonite using a Perkin Elmer Spectrum BX Infrared spectrometer with the wave range of 4000–400 cm^−1^ and the technique of KBr pellet. Four scan replicates were performed for each sample to ensure reproducibility and uniformity.

### Batch experimental procedures

Preparation of stock solution of IP and OP: The stock solution of IP was prepared via dissolving 1.47 g of potassium dihydrogen phosphate (KH_2_PO_4_) in 1 L of deionized water to prepare 1000 mg\L. Then, the other tested solutions were prepared by diluting the stock solution to desired concentrations (1, 5, 10, 15, 20 mg/L) of (IP), while the malathion compound was used to prepare the organic phosphorus (OP) by diluted with deionized. Both forms of (P) were determined using DR 3900 Spectrophotometers^29^.

The batch experiment: Selection of the optimum conditions of using natural and Na-bentonite to remove the selected pollutants. The batch approach was employed to measure phosphors uptakes on the adsorbents conducted in 250 mL Erlenmeyer flasks. The adsorption properties of natural and activated bentonite were studied under various conditions, including pH values (2, 6, 8, 10 with negligible volumes of sodium hydroxide (NaOH) or hydrochloric acid (HCl) were added to the solution system to adjust the desired pH), sorption time (from 5 to 120 min.), adsorbent dosage (0.5, 1, 1.5, 2 g) and initial concentration of IP and OP (1, 5, 10, 15, 20 mg/L). Batch sorption studies were carried out in 50 ml polyethylene filled with 20 ml of different concentrations from phosphors solution, at constant temperature (25 °C). The mixture in 250 mL Erlenmeyer flasks was kept on a constant agitation shaker (200 rpm at 25 °C) to enhance the interaction of the adsorbent and phosphors solution.

Samples were taken from the solution in predetermined intervals during the stirring process to determine the phosphate concentration remaining in the medium. Before the analysis, the samples were filtered through a 0.45 membrane syringe filter and analyzed for the remaining supernatant fluid phosphate amount. It was observed that the absorbed P and absorbent reached equilibrium after 40 min. However, the experiment has been running for 120 min to ensure that the absorption process reached complete equilibrium. At the end of the experiment, the adsorbents were removed from suspension by centrifuging at 4000 rpm for 20 min and the residual total IP and OP concentration in the solutions was measured using a UV–visible spectrophotometer (Hack 3900 model). All experiments were repeated twice, and average values were used for future calculations. The adsorption efficiency related to the amount of phosphorous adsorbed was determined by Eq. (2); Where, *C*_*∘*_ is the initial concentration of pollutants (mg/L) and C_e_ is the final concentration of both forms of phosphors after adsorption (mg/L).

2 $$Removal \,Efficiency \left( {RE\% } \right) = \frac{{\left( {C_{{{\text{o}} - }} C_{e } } \right)}}{{C_{{\text{o}}} }} \times 100$$

Water analyses: The water samples preservation and analyses regarding all investigated parameters were conducted according to the Standard Methods for the Examination of Water and Wastewater^30^.

### Quality control

The quality control samples for organic and inorganic phosphorus (2.5, 7.5) were prepared and measured, while the calibration curve for phosphorus analysis was constructed with a blank and seven standards (Merck Germany). The accuracy and precision of the metal measurement were confirmed using external reference material for phosphorous in the water.

### Regeneration of Adsorbents

Regeneration of the spent adsorbents (natural and Na-bentonite) was done by two chemical substances for comparison. The first substance was 1 M KCl^8^; the two adsorbents were washed gently to remove ions from their surface, then with deionized water to remove any residual, then dried at 80 °C, and left to cool in a desiccator. For the second substance; the same previous method was applied to the spent materials, but with a neutral solution from 5 μM Ethylenedinitrilotetraacetic acid disodium salt dihydrate (Na2-EDTA). A repeat experiment was carried out for comparison of the original and the regenerated adsorbents on wastewater treatment (3-trials).

## Results and discussion

### Characterizations of natural and activated bentonite (Na-bentonite)

**Figure 1:**
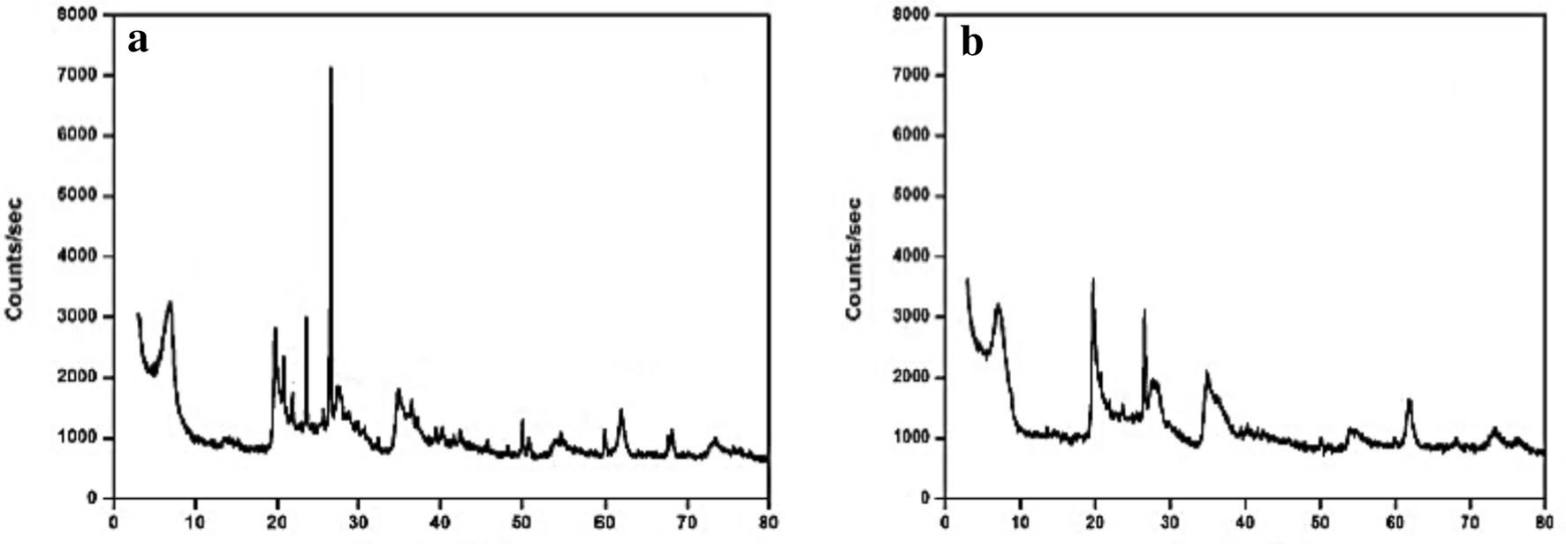
XRD pattern (**a**) natural bentonite (**b**) Na-bentonite.

The X-ray diffraction patterns of natural and Na-bentonite samples were presented in Fig. 1. The XRD pattern of natural bentonite revealed that the major mineral was montmorillonite, with quartz, kaolinite, and dolomite^8^, (Table 1). The patterns of Na-bentonite revealed that the major mineral is Na-montmorillonite; with minor amounts of kaolinite, and dolomite also present^31^. The results proved that the natural bentonite contained quartz, while the Na-bentonite lacked it (Table 1).

**Table 1:** Mineralogical composition, mass (%) of adsorbents.

Composition natural bentonite (wt%)	Composition Na-bentonite (wt%)
Quartz (Si_3_O_6_)	Na-montmorillonite (Na, Ca_0.33_, Al, Mg_2_Si_4_, O_10_, OH_2_·nH_2_O)
Montmorillonite (Si_7.80_Al_1.72_Li_0.16_Fe_0.20_Mg_0.28_O_20_)	
Kaolinite (Al_2_Si_2_O_9_H_4_)	Kaolinite (Al_2_Si_2_O_9_H_4_)
Dolomite (Ca_3_Mg_3_C_6_O_18_)	Dolomite (Ca_3_Mg_3_C_6_O_18_)

The XRD data show that adding sodium carbonate causes changes or modifications in the minerals structure of the bentonite, as seen in the Na-bentonite patterns (Na_2_CO_3_) (Fig. 1). The activated sample lacked quartz, whereas the spectrum peaks of a little amount of quartz mineral were decreased. The spectral peaks of the primary mineral montmorillonite were visible. Two significant crystalline sod-carbonate diffraction lines (55 and 35 A) were present, indicating crystalline sod-carbonate buildup^32^.

X-ray data of Na-bentonite exhibit a significant shift or displacement in the position of a few spectrum peaks, such as 31.2–26.6 A. The sodium ions were absorbed to the edge surface of the montmorillonite minerals after sod-carbonate addition, forming a hydrated shell^33^. The prior finding indicated that the tetrahedral and octahedral sheets have disintegrated, and the structural cations have been released, indicating that these cations have been removed from the octahedral positional structure and replaced by sodium ions. The interlamellar gap between crystals is squeezed^34^. The chemical composition and interior structure of activated Na-bentonite clay were altered. XRD pattern analysis, in particular, shows that natural bentonite's adsorptive ability has increased. The peak intensity decreased due to the activation process. This mostly found in the montmorillonite minerals, which means a reduction in its composition and also the elimination of the impurity of quartz content.

Furthermore, the XRD peaks patterns of natural bentonite showed relative symmetry, whereas the diffraction peak of Na-bentonite clay showed more dissymmetry. On the other hand, the presence of some peaks splitting proved the crystallization processed to a lower similarity or partial deformation of its mineral structure, showing that small vibrations that can frequently be seen in peak broadening. Such properties seem to show that bentonite was easily activated or medicated by sodium. The reduction in some intensity with width increase to another spectrum peaks at 24.1 clearly shows that the activation process has significantly affected the bentonite clay crystalline structure; hence the clay crystal lattice structure is decaying, recommending that the activation process was associated with the presence of an amorphous state, as revealed by IR results.

The minerals compositions of both adsorbents have been determined using XRF as displayed in Table 2. These results reported that the main composing elements were Si, O, and Al, which confirmed that the aluminosilicate clay is used in this investigation. The lower of K, Mg, Fe, and Na can be considered as exchangeable cations within the bentonite clay structure.

**Table 2:** XRF elemental composition (mass %) of adsorbents.

Content	Composition (wt%)	
	Natural bentonite	Na-bentonite
Al_2_O_3_	22.64	19.34
SiO_2_	52.22	51.64
Fe_2_O_3_	4.77	5.78
CaO	2.85	1.71
Na_2_O	1.42	3.12
K_2_O	1.39	1.13
MgO	3.54	2.98
TiO_2_	0.41	1.08
P_2_O_5_	0.2	0.17
LOI	10.98	14.20

The high cation exchange capacity is a well-known feature of this clay, and therefore, these last elements can be exchanged by others to tailor the bentonite composition for the intended application. In this case, Na has been implemented using sodium carbonate as a source of cation. After treatment, the amount of sodium increased from 1.24 to 3.12%, while potassium and magnesium amounts along with some alumina content was consequently decreased, pointing out that Na-bentonite has been successfully exchanged with cations of sodium. The quantity of Na loaded in bentonite is somewhat less than the one studied by Harrane^35^ for montmorillonite where a sodium content increasing from 1.35 to 3.26% was achieved. The sodium content value (3.12%) recorded for Na-bentonite shown in Table 2 was similar to those previously reported in the literature^36^ where sodium content between 2.9 and 3.2% was found by these three researchers after sodium treatment of the Algerian bentonite clay.

Table 3 shows the surface area and particle size distribution for both adsorbents. The obtained results revealed that the surface area analysis recorded 74.21, 138.51 m^2^g^−1^ which indicated a large surface area for Na-bentonite than a natural sample, led to more available active sites. The results showed that the surface area increased with increasing of sodium on the surface of bentonite clay. The improved surface area indicated the number of active sites increased on the surface of the adsorbent, which improved the enhancement in adsorption efficiency^37^. While the distribution of particle size for natural and Na-bentonite was less than 2 µm which recorded 78.23 and 84.1% respectively.

**Table 3:** Particle size distribution of natural and natural and Na-bentonite.

Adsorbent clay	Surface area (m^2^/g)	Particle size distribution (%)		
		Clay < 2 µm	Silt 63–2 µm	Sand > 63 µm
Natural	74.21	78.23	15.07	6.70
Na-bentonite	138.51	82.09	13.88	4.03

**Figure 2:**
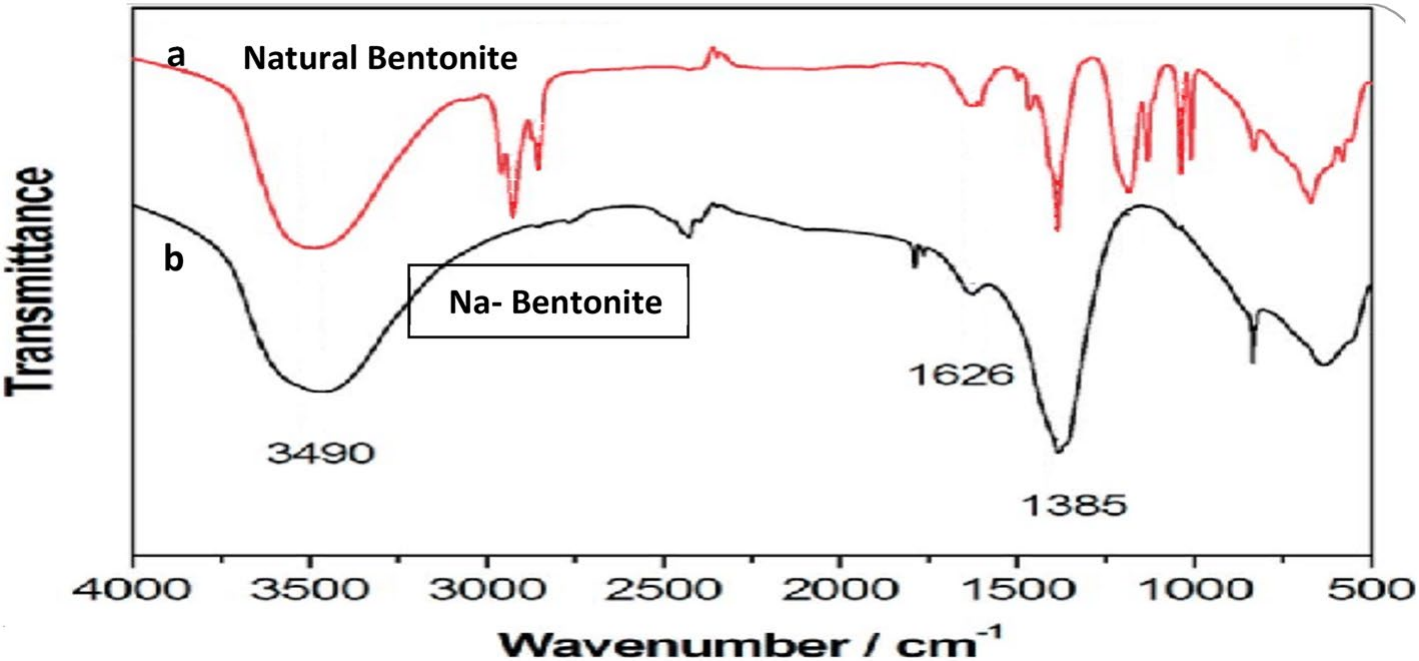
Infrared (IR) spectra of bentonite (**a**)- natural bentonite and (**b**)- Na-bentonite).

Figure 2 displays FTIR analysis of natural and Na-bentonite in order to determine the functional groups on the surface of bentonite that are responsible for adsorption and to explore the influence of sodium activation on its chemical composition. The 550–400 cm^−1^ area of the natural bentonite spectrum contained absorption bands caused by bending vibrations of Si–O groups. The bands owing to Si–O–Al and Si–O-Side formations appeared near 530 and 460 cm^−^1, respectively, according to the spectra^38^. Quartz was also responsible for bands at 706,800, and 2355 cm–1 in the spectrum ^39^. A large complex band near 1030 cm^−1^ was caused by stretching vibrations of Si O groups^40^. The 1641 cm^−1^ peak, on the other hand, was for H–O–H bending and the stretching vibration of OH^−^.

The bending of H–O–H had a peak at 1641 cm^−1^, and hydroxyl (OH^-^) vibration stretching which appeared around 3451 cm^−1^. The adsorption spectrum band at 3616 cm^−1^ was attributed to the vibrations stretching of hydroxyl (OH^−^) dioctahedral groups in the bentonite clay. The most noticeable significant changes after sodium activation were a decline in the spectrum band of Si–O stretching intensity at the region 1030 cm^−1^. The above changes indicate the formation of three-dimensional networks of amorphous silica due to the activation process, potentially exposing more porous structures and causing damage formation to the tetrahedral layer. The strength of the bending and stretching bands that described the octahedral sheet reduced by 1641 cm^−1^ for Al–Al–OH, demonstrating that the octahedral layer was damaged. However, as water and the group of hydroxyl groups attached to the octahedral structure cations were lost, the absorption spectrum ascribed to the hydroxyl (OH^-^) vibration around 3616 cm^−1^^41^ dropped dramatically. The drop in the distinct spectrum band was around 3451 cm^−1^ indicated the basic stretching vibrations for various (OH^−^) groups found in Fe–OH–Al, Al–OH–Al, and Mg–OH Al in the octahedral layer^42^, verifying the layer’s disfiguration. There was a decrease of the spectrum band around 2355 cm^−1^ and a reduction in the intensities of spectrum bands near 706, 800 cm^−1^, showing that the activation treatment improved the adsorptive characteristics of the adsorbent. During the activation treatment, the majority of the band positions remained unchanged, showing that the main adsorbent structure was not changed.

**Figure 3:**
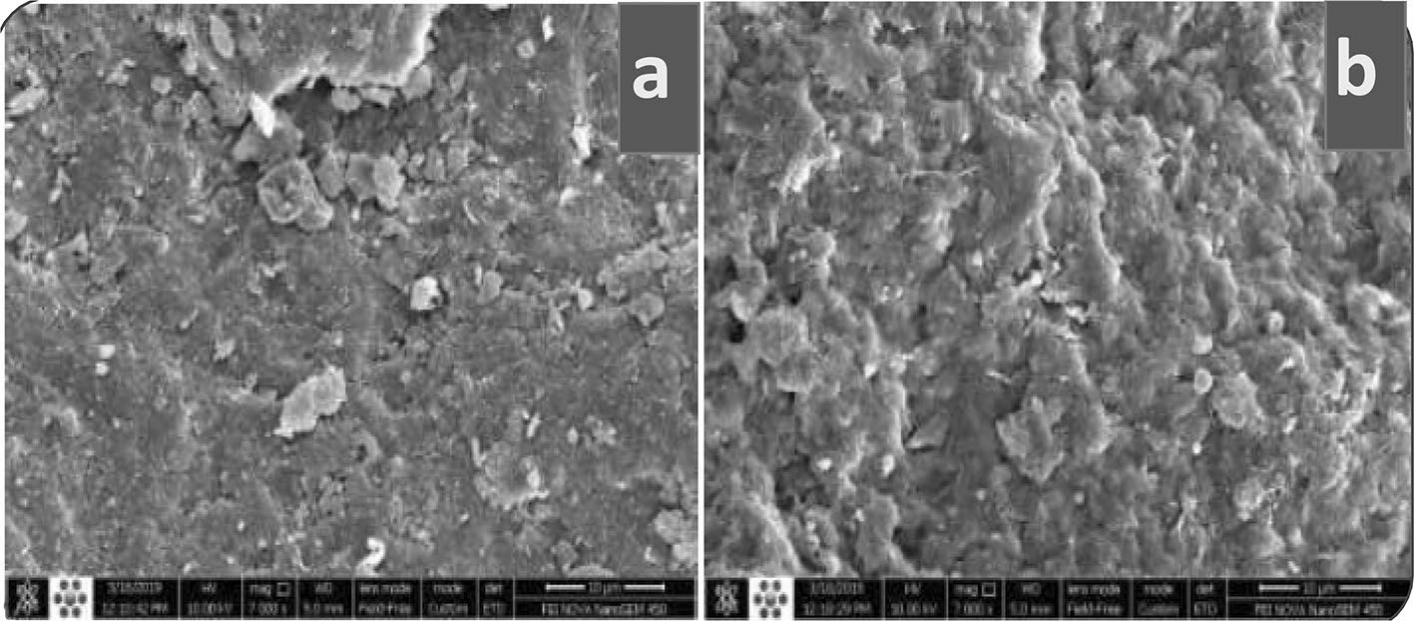
SEM images of (**a**) Natural bentonite (**b**) Na-bentonite.

Figure 3 shows the electro-scanning microscope (ESM) of natural and Na-bentonite samples, which showed the surface of natural bentonite was relatively flatter and smoother than activated bentonite with sodium treatments, while the surface morphology of activated Na-bentonite has been porous like structure with rough appearance and infrequent, dispersed block structure at different sizes and look like an ultra-fine thin layer. This appearance may be due to the reduction in some certain amorphous phases which were typically connected with the treatment of natural bentonite with sodium^43^.

### Batch study

There are many factors that affect the sorption of toxic materials by clays such as pH of the solution, the weight of adsorbent, initial concentrations of pollutants, temperature, and contact time. The removal efficiency of natural bentonite towards different organic and inorganic pollutants was studied under the previous conditions except for temperature.

### Effect of pH of the solution

The pH parameter of the aqueous solution is an important controlling parameter in sorption processes^44^. The anion exchange capacity is strongly controlled by the pH of the solution and by the surface chemistry of the clays^45^. Tables 4, 5, 6 and 7 display the removal efficiency of both forms of phosphorus (IP and OP) from aqueous solutions by the studied adsorbents at pH 2–10. The outcomes revealed that the removal percentage of both forms of phosphorus onto natural and Na-bentonite increased with increasing of pH value from 2 to 6 with higher removal efficiency recorded at pH equal to 6 with removal efficiency 85%, 42% and 81%, 38% for IP and OP, respectively. The high adsorption observed for activated bentonite at lower pH (at strongly acidic conditions) may be related to the electrostatic attraction between the positive charge on the surface of the adsorbent and the negative charge on anionic adsorbate (i.e., PO_3_^-^, etc.)^46^. While the low adsorption at high pH returns to the competition with OH^-^ ions with phosphorous that may reduce phosphate (PO_4_^3−^) bonding capacity on adsorbents surface ^47^. Our results found support by Gupta and Bhattacharyya^47^ observed the maximum phosphorus sorption was greater in kaolin when pH changed from 4.3 to 6.3.

**Table 4:** Variation of removal efficiency (%) of phosphors (IP, OP) at different pH levels and 0.5 g Natural (NB) and Na-bentonite (Na-NB).

pH values	Removal efficiency of activated bentonite (Na-B) to different IP concentration in relation with pH by 0.5 g Natural and Na-bentonite																			
	1 mg/L				5 mg/L				10 mg/L				15 mg/L				20 mg/L			
	IP		OP		IP		OP		IP		OP		IP		OP		IP		OP	
	NB	Na-B	NB	Na-B	NB	Na-B	NB	Na-B	NB	Na-B	NB	Na-B	NB	Na-B	NB	Na-B	NB	Na-B	NB	Na-B
2	7.0	22.0	4.0	19.0	8.0	13.2	4.4	5.2	4.2	10.2	2.5	7.9	5.3	10.0	1.6	1.9	2.5	11.0	1.3	8.0
4	19.0	38.0	13.0	32.0	18.0	28.6	15.2	19.8	10.2	21.2	8.8	15.7	8.6	14.2	5.1	9.1	5.5	23.8	4.2	19.0
6	27.0	60.0	22.0	49.0	22.4	53.0	20.2	47.2	21.8	52.0	19.9	44.0	19.3	37.3	17.1	35.7	13.5	33.3	11.0	27.8
8	26.0	58.0	19.0	42.0	22.2	52.8	15.8	44.4	21.6	51.1	16.6	41.2	18.0	35.5	16.2	34.4	13.3	32.0	9.4	24.0
10	26.0	58.0	18.5	42.0	22.4	53.0	16.2	44.2	21.6	51.8	16.8	40.8	17.9	24.7	16.2	35.3	13.1	32.0	9.9	23.9

**Table 5:** Variation of removal efficiency (%) of phosphors (IP, OP) at different pH levels and 1 g Natural (NB) and Na-bentonite (Na-NB).

pH values	Removal efficiency of activated bentonite (Na-B) to different IP concentration in relation with pH by 1 g Natural and Na-bentonite																			
	1 mg/L				5 mg/L				10 mg/L				15 mg/L				20 mg/L			
	IP		OP		IP		OP		IP		OP		IP		OP		IP		OP	
	NB	Na-B	NB	Na-B	NB	Na-B	NB	Na-B	NB	Na-B	NB	Na-B	NB	Na-B	NB	Na-B	NB	Na-B	NB	Na-B
2	6.0	11.0	4.0	6.0	8.4	14.0	4.4	9.8	3.9	11.3	2.5	4.7	5.1	18.0	2.6	6.2	3.9	29.0	3.9	14.8
4	17.0	29.0	13.0	20.0	16.8	22.8	13.8	19.6	14.3	29.0	13.3	15.9	7.5	28.4	5.3	9.9	7.8	30.8	7.8	9.5
6	32.0	59.0	31.0	63.0	29.2	56.4	27.8	51.6	25.8	53.8	24.2	50.7	24.0	40.7	20.1	39.9	16.3	36.0	14.9	34.5
8	31.0	68.0	25.0	58.0	29.0	56.2	25.0	48.8	25.7	53.7	23.1	48.8	23.0	39.9	19.3	36.5	15.8	35.5	14.0	32.4
10	31.0	68.0	28.0	59.0	29.0	56.2	25.2	50.0	25.7	53.8	23.5	48.2	22.9	39.9	19.7	36.5	15.8	35.5	13.9	32.4

**Table 6:** Variation of removal efficiency (%) of phosphors (IP, OP) at different pH levels and 1.5 g Natural(NB) and Na-bentonite (Na-B).

pH values	Removal efficiency of activated bentonite (Na-B) to different IP concentration in relation with pH by 1.5 g Natural and Na-bentonite																			
	1 mg/L				5 mg/L				10 mg/L				15 mg/L				20 mg/L			
	IP		OP		IP		OP		IP		OP		IP		OP		IP		OP	
	NB	Na-B	NB	Na-B	NB	Na-B	NB	Na-B	NB	Na-B	NB	Na-B	NB	Na-B	NB	Na-B	NB	Na-B	NB	Na-B
2	7.0	16.0	6.0	14.0	6.6	19.0	4.8	15.2	3.3	11.6	2.7	9.0	3.3	5.0	2.2	3.7	3.8	8.8	2.8	4.6
4	16.0	32.0	12.0	26.0	16.0	23.4	13.4	18.0	15.3	31.3	13.6	17.4	8.8	8.4	7.9	14.3	6.7	15.8	6.4	8.8
6	38.0	78.0	31.9	72.0	30.0	63.6	27.2	61.6	26.6	57.8	21.6	53.0	24.7	47.3	19.7	45.9	19.4	42.0	13.9	39.5
8	37.0	77.0	24.8	65.0	29.4	63.4	25.2	55.6	26.5	57.6	20.8	49.0	24.0	47.0	18.4	44.4	38.8	41.4	12.4	38.4
10	37.0	78.0	24.5	66.0	29.0	63.4	25.4	58.0	26.6	57.8	20.7	49.8	23.9	46.5	18.1	44.3	18.8	41.1	12.3	38.4

**Table 7:** Variation of removal efficiency (%) of phosphors (IP, OP) at different pH levels and 2 g Natural (NB) and Na-bentonite (Na-B).

pH values	Removal efficiency of activated bentonite (Na-B) to different IP concentration in relation with pH by 2 g Natural and Na-bentonite																			
	1 mg/L				5 mg/L				10 mg/L				15 mg/L				20 mg/L			
	IP		OP		IP		OP		IP		OP		IP		OP		IP		OP	
	NB		NB	Na-B	NB	Na-B	NB	Na-B	NB	Na-B	NB	Na-B	NB	Na-B	NB	Na-B	NB	Na-B	NB	Na-B
2	6.20	18.0	5.0	13.0	11.8	19.2	9.6	15.2	4.2	12.7	3.7	10.6	3.1	5.3	2.1	5.2	4.0	7.4	2.0	6.1
4	20.0	37.0	13.0	24.0	19.0	26.6	17.6	23.2	17.7	32.8	15.4	27.6	7.7	14.2	6.9	14.1	7.3	28.2	6.1	22.4
6	42.0	85.0	38.0	81.6	31.2	69.6	22.6	67.2	28.2	61.2	21.6	60.3	22.0	51.9	19.3	48.6	20.5	42.0	17.7	44.9
8	41.0	83.0	32.0	76.0	30.4	69.6	18.0	64.6	26.6	61.1	20.1	58.8	23.0	51.8	16.9	47.7	19.5	41.0	16.3	43.2
10	41.0	84.0	33.0	75.0	30.4	69.6	17.8	65.0	26.5	61.1	20.5	59.0	22.9	51.7	17.0	47.7	19.5	41.0	16.3	43.3

### Effect of adsorbents doses on equilibrium

The effect of studied adsorbents dose on the removal efficiency percentage of both phosphorus forms in solution was investigated by natural and Na-bentonite dose (0.5–1.0–1.5–2.0 g), as shown in Table 8. The obtained data of the current study showed that the removal percentage of IP and OP was high in the case of Na-bentonite compared with natural bentonite and increased with the increasing of adsorbents dose. The removal percentage was recorded 42%, 85% and 38%, 81.6% for IP and OP by 2 g from natural, and Na-bentonite, respectively. This is due to, metal ions were competing for limited adsorption sites at a lower dose, but as the dose increased, there was a larger surface area with more available vacant sites that in turn increased the adsorption percentage^48^. The obtained results are similar to the observations were found by Gupta and Bhattacharyya^47^.

**Table 8:** Removal efficiency (%) with different adsorbents weight and phosphorous concentrations.

Phosphorous conc. mg/L	Removal efficiency of initial phosphors concentration with wt. of Natural (NB) and activated bentonite (Na-B)															
	0.5 g				1 g				1.5 g				2 g			
	IP		OP		IP		OP		IP		OP		IP		OP	
	NB	Na-B	NB	Na-B	NB	Na-B	NB	Na-B	NB	Na-B	NB	Na-B	NB	Na-B	NB	Na-B
1	27.0	60.0	22.0	49.0	32.0	68.0	31.0	63.0	38.0	78.0	31.9	72.0	42.0	85.0	38.0	81.6
5	22.4	53.0	20.2	47.2	29.2	56.4	27.8	51.6	30.0	63.6	27.2	61.8	31.2	69.6	22.6	67.2
10	21.8	51.0	19.9	44.0	25.8	53.8	24.2	50.7	26.6	57.8	21.6	53.0	28.2	61.2	21.6	60.3
15	19.3	37.3	17.1	35.7	24.0	40.7	20.1	39.9	24.7	47.3	19.7	45.9	24.0	51.9	19.3	48.6
20	13.5	32.5	11.0	27.8	16.3	36.0	14.9	34.5	19.4	42.0	13.9	39.5	20.5	47.8	17.7	44.9

### Effect of contact time on phosphorus removal and kinetic studies

**Figure 4:**
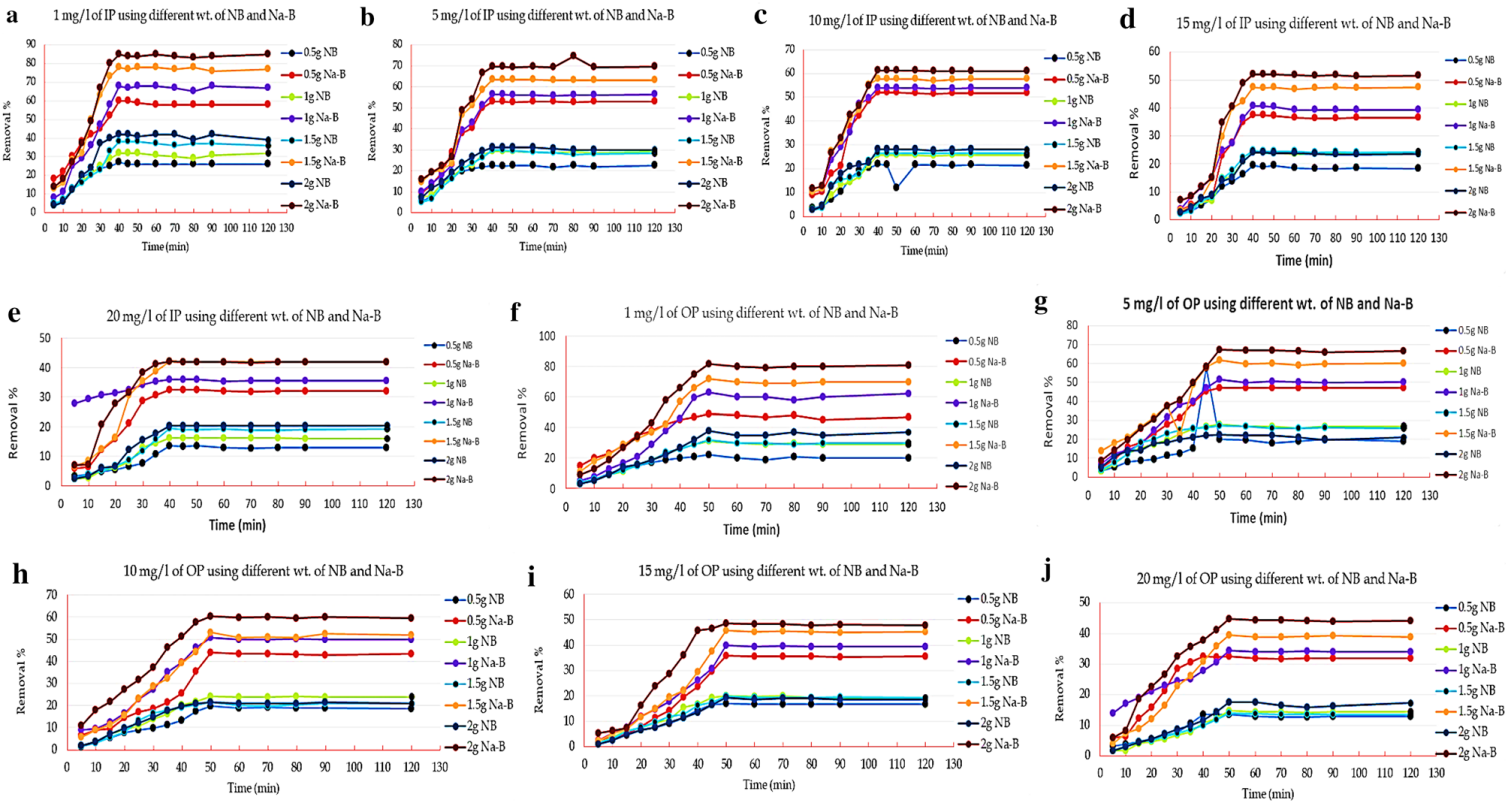
(**a–j**) Effect of contact time on adsorption of different weights from IP and OP by natural (NB) and Na-bentonite (Na-B).

The contact time is considered one of many factors that influence the adsorption of metals onto adsorbents^49^. The removal percentage of both IP and OP by adsorbents was examined at various time intervals (Fig. 4a–j). The adsorption pattern of phosphors has gradually increased with time increases until 40 and 50 min for both IP and OP on the surface of adsorbents, then the removal percent became constant (i.e. equilibrium is attained). The data revealed that the adsorption was fast at the beginning of the experiment, with an observed high removal percentage for Na-bentonite compared with natural form and gradually the adsorption became slower until the equilibrium was attained; this could be related to the availability of the Na-bentonite surface area, a large number of vacant binding sites, more than natural bentonite and the well-oriented functional groups during the initial stages so the interaction between the Na- bentonite and both forms of phosphorus in solution was feasible.

Our outcomes found support from Elsayed et al.^8^ and Chitrakar et al.^50^, they observed that the adsorption of total phosphorus (TP) and heavy metals pollutants to bentonite column increased with increasing time. The previous case can be clarified as, with the increase in contact time, the adsorbent’s surface became gradually linked and covered with phosphorus ions^51^. So, the occupation of the other remaining unoccupied surface sites is difficult due to the repulsion forces between the ions in the clay surface and liquid phases. The relative equilibrium of contact time (40, 50 min in case of IP and OP respectively) may also reveal an economic advantage if it applicated on the large-scale experiment to wastewater treatment systems^52^. Such results are promising by the economic viability because of shorter treatment time helps in the reduction of capital and operational costs because longer shaking time consumes more energy, and consequently the cost increases^53^. Thus, as referenced by Cucarella and Renman^54^ relying upon the type of adsorbent utilized, adsorbent and solution percent, phosphors concentration, temperature and agitation, the equilibrium could be after minutes, hours, days, or even months^55^.

**Figure 5:**
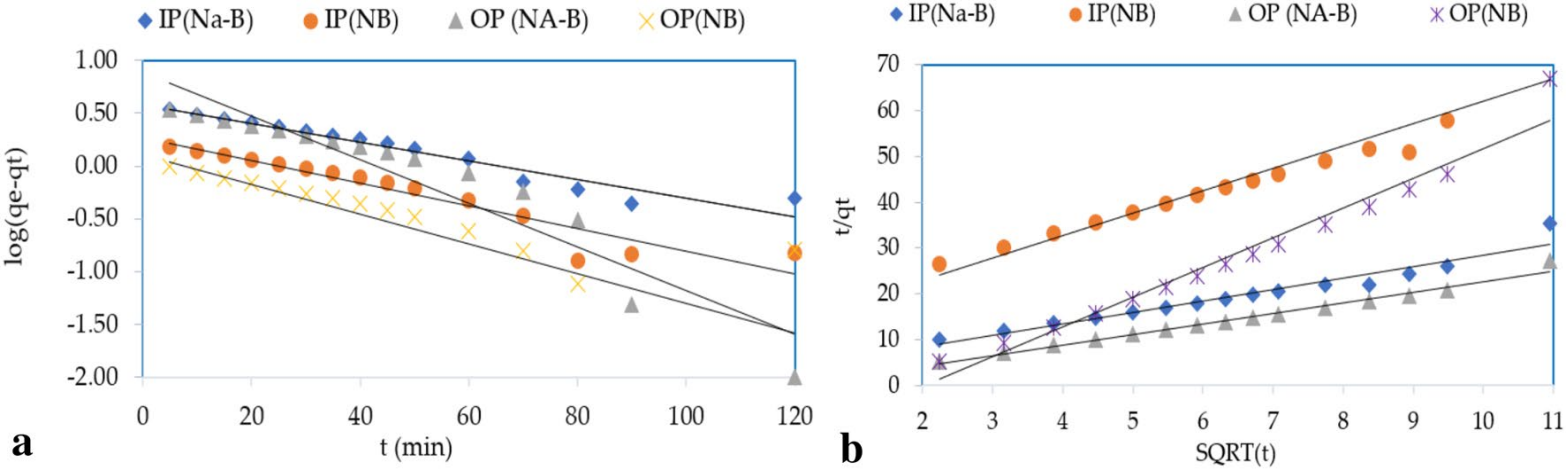
(**a**) Pseudo-first-order and (**b**) Pseudo-second-order adsorption kinetics.

Kinetic studies are carried out to acquire information on the physical nature of processes occurring in IP and OP sorption. Table 9 and Fig. 5 present parameters for kinetic models (Pseudo-first, and Pseudo-second-order) for concentrations of 1 mg/L IP and 2 mg/L OP mg/L, using NB and Na-B, respectively. The pseudo-first-order kinetic equation states that the removal of IP and OP ions from the solution over time is proportional to the difference between the sorption volume of the NB & Na-B and the measure of IP and OP ions after a specified time (t) in equilibrium. Depending in the correlation coefficient R^2^ value; the first-order kinetics model is not definitely a reasonable model portraying the sorption process occurring in that investigation and the best-fitted model is the pseudo-second-order kinetics model because of the high value of correlation coefficient R^2^ for both materials (NB and Na-B). This kinetic model recognizes that ions are removed from the aqueous media by physicochemical interactions. The model of this kinetics is based on chemical sorption^56^.

**Table 9:** Isotherm parameters of equilibrium of IP and OP on Natural bentonite (NB) and sodium-bentonite (Na-B).

	Pseudo-first-order			Pseudo-second-order		
	q1(mg/g)	K1 (1/h)	R2	q2(mg/g)	k2(g/mg/h)	R2
IP(Na-B)	3.75	0.0020	0.91	0.4004	1.9	0.94
IP(NB)	1.85	0.0246	0.90	0.2040	1.8	0.92
OP (NA-B)	7.73	0.0477	0.89	0.4303	− 9.2	0.98
OP(NB)	1.27	0.0322	0.54	0.1543	− 3.2	0.96

### Effect of phosphors initial concentration and adsorption studies

The obtained data indicate that the sorption capacity of natural and Na-bentonite to both forms of phosphors is affected by their initial concentrations as presented in Table 9. The data revealed that, the removal efficiency of adsorbents was increased as the initial concentrations of phosphors decreased at 20 mg/L. The data showed the removal percentage recorded 42%, 85% and 38%, 81.6% for IP and OP by 2 g of natural, and Na-bentonite, respectively. The outcomes of the current study agreed with Zhang et al.^57^. They reported the sorption of natural bentonite to various metal ions was increased by reducing the initial concentrations of pollutants.

Four isotherm models were used for the equilibrium modeling, the first is Langmuir model^58^, which suggests monolayer adsorption and is expressed by Eq. (3), while the second is Freundlich isotherm^59^ model, which is presented by Eq. (4), but the third is Temkin model^60^, which is described by Eq. (5) and the fourth model is Dubinin-Radushkevich model^61^, which is characterized by Eq. (6):

3 $$\frac{{C_{e} }}{{q_{e} }} = \frac{1}{{q_{max} }} b + \frac{{C_{e} }}{{q_{max} }}$$

4 $$lnq_{e} = \ln K_{f} + \frac{1}{n} ln C_{e}$$

5 $$q_{e} = BlnA_{T} + BlnC_{e}$$

6 $$lnq_{e} = lnq_{s} - K_{DR} \varepsilon^{2} , where\, \varepsilon = RT ln\left[ {1 + \frac{1}{{C_{e} }}} \right]$$

where Ce is metal ions concentration at equilibrium (mg/L), qe is amount of metal adsorbed at equilibrium (mg/g), q_max_ is maximum adsorption capacity of the sorbent (mg/g), b is the Langmuir adsorption constant (L/mg), K*f* and n are Freundlich constants that include factors that impact adsorption capacity and adsorption intensity, B (g/mg/h^2^) and A_T_ (mg/g/h^2^) are Temkin constant related to the heat of sorption and maximum binding energy, R represent the gas constant (8.314 J/mol K), T absolute temperature (K), qs = theoretical isotherm saturation capacity (mg/g); K_RD_ is Dubinin–Radushkevich isotherm constant (mol^2^/kJ^2^)respectively.

**Figure 6:**
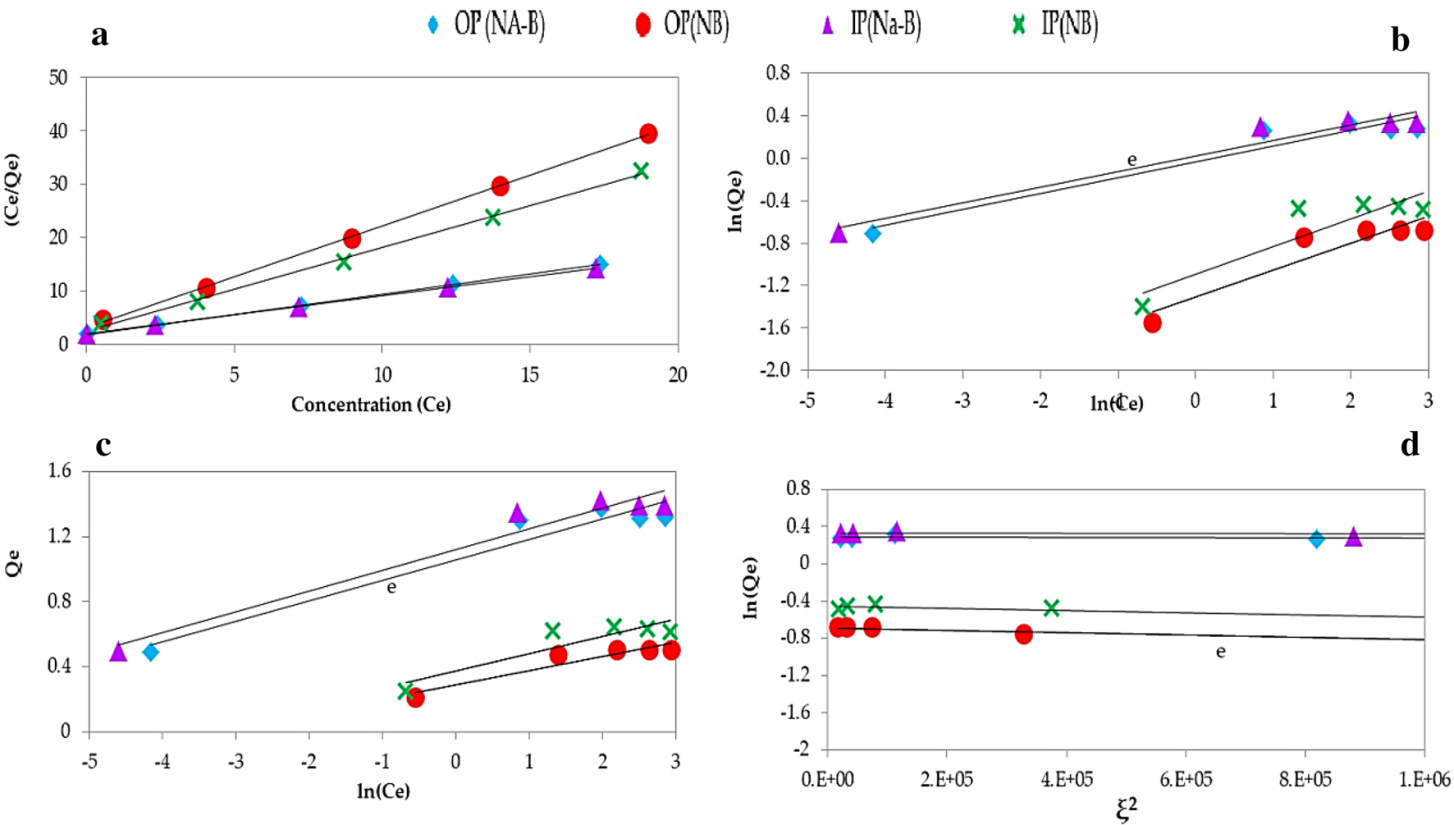
Adsorption isotherm models where: (**a**) Langmuir, (**b**) Freundlich, (**c**) Temkin, and (**d**) Dubinin-Radushkevich.

Four adsorption isotherms (Freundlich, Langmuir, Temkin, and Dubinin-Radushkevich) were investigated for adsorption of IP, and OP on NA-B and NB in this investigation (Table 10) and Fig. 6a–d). The regression coefficient (*R*^2^) was evaluated to differentiate between the four isotherms models (Table 10). The *R*^2^ value was close to unity in the case for the Langmuir model (Freundlich: 0.82–0.94, Langmuir: 0.999, Temkin: 0.82–0.95, and Dubinin-Radushkevich: 0.97–0.98). *R*^2^ value was found satisfactory showing fitness with the Langmuir isotherm model for IP, and OP on NA-B and NB. If value of *b* is in between 0 and 1, the system is considered suitable for adsorption purpose, for the present study value of *b* ranged between 0.36 and 0.62 indicating the IP, and OP are suitable for NA-B and NB adsorption. The theory of Langmuir isotherm coincides with Pseudo-second-order outcomes that are fitted to the sorption occurring in the experiment. In the first step, the surface adsorption process was associated with many active sites thus the reaction was quick and natural. Increasing the concentration of IP and OP was the cause of the increased speed of the sorption process due to the greater number of collisions between the IP and OP and the NB and Na-B^62^.

**Table 10:** Adsorption isotherm parameters.

Parameters	Langmuir model				Freundlich model		
	q_max_	b	R*L*	R^2^	Kf	n	R^2^
OP (NA-B)	1.32	0.38	0.12–0.72	0.999	0.96	6.70	0.93
OP(NB)	0.53	0.60	0.08–0.62	0.999	0.27	3.94	0.87
IP(Na-B)	1.40	0.36	0.07–0.74	0.999	1.02	6.82	0.94
IP(NB)	0.64	0.62	0.07–0.62	0.998	0.34	3.83	0.82

	Temkin model			Dubinin-Radushkevich model			
	A_T_	B	R^2^	qs	K_DR_	E	R^2^
OP (NA-B)	4361.4	0.1258	0.94	1.33	8 × 10^–9^		0.997
OP(NB)	28.9	0.0863	0.89	0.50	1 × 10^–7^		0.998
IP(Na-B)	6318.6	0.1275	0.95	1.39	7 × 10^–9^		0.998
IP(NB)	32.6	0.1066	0.82	0.64	1 × 10^–7^		0.997

The fitting results show that the equilibrium constant RL of the Langmuir equation is 0 < RL < 1, so that the adsorption with that order IP (NB) > OP(NB) > IP(Na-B) > OP(Na-B). A typical Langmuir isotherm shows a characteristic horizontal asymptote indicating saturation after the monolayer adsorption. This indicated that the adsorption mechanism of IP, and OP on Na-B and NB was chemisorption.

### Effect of temperature on phosphors removal and thermodynamic studies

**Figure 7:**
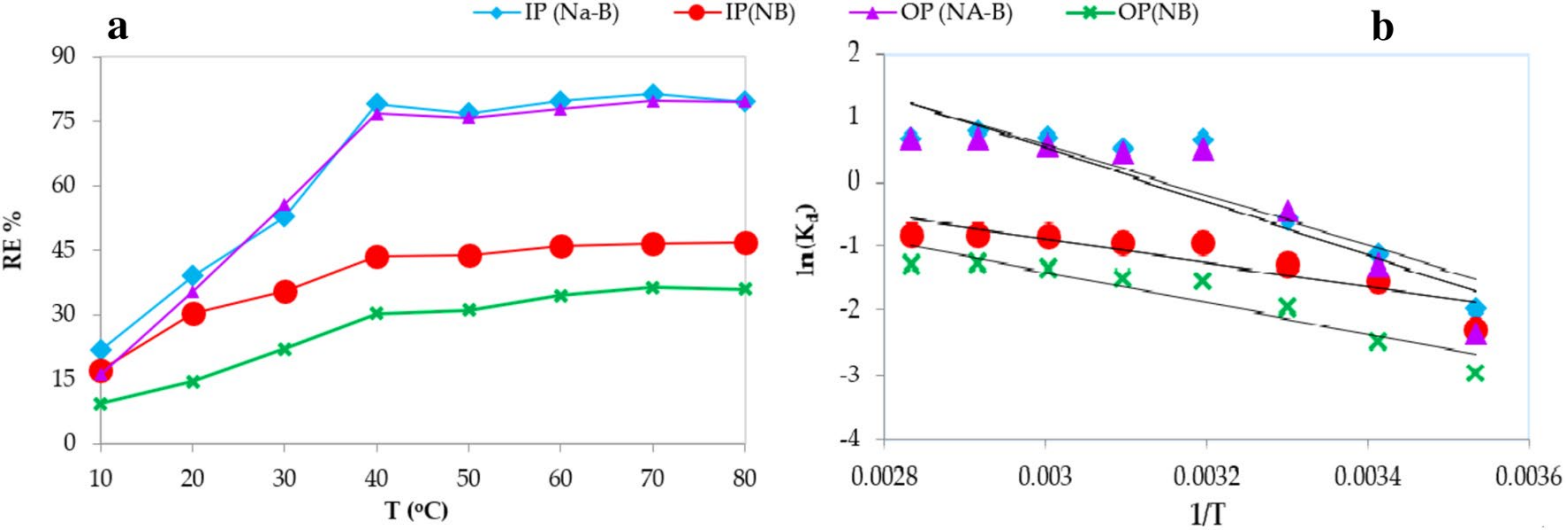
The change in removal efficiency (RE%) with temperature (**a**), while (**b**) is ln(K_d_) versus 1/T.

As evident from Fig. 7a the maximum adsorption yield was determined as 97.2% at 40 °C temperature and in 1 mg/L concentration IP, and OP by natural bentonite (NB), and activated bentonite (Na-B).

Thermodynamic parameters for the biosorption process such as free energy change (ΔG^0^), enthalpy changes (ΔH^0^), and entropy change (ΔS^0^) can be estimated through following Eqs. (7–9)^63^ where R = universal gas constant, 8.314 J\mol K; T = absolute temperature (K). K_d_ is equilibrium constant. The changes in enthalpy and entropy for the respective biosorbents were obtained from the Van’t Hoff plot of ln K against 1/T, Eq. (8), Fig. 7b.

7 $$- \Delta G^{0} = - RT ln K_{d}$$

8 $$ln K_{d} = \frac{{\Delta H^{0} }}{RT} + \frac{{\Delta S^{0} }}{R}$$

9 $${\Delta }G^{0} = {\Delta H}^{0} - T{\Delta S}^{0}$$

The estimated values of ΔH°, ΔS°, and ΔG° are shown in Table 11. The negative free energy values (ΔG°) show the suitability of the process and spontaneous nature of adsorption, while the negative values of enthalpy (ΔH°) indicate the system involves exothermic physicochemical adsorption in nature. The positive ΔS° values indicate the increase in the randomness in of the system with increase in temperature. The enthalpy-entropy compensation is well illustrated through the corresponding values obtained for the bio-sorbents. The adsorption process is more exothermic in OP(Na-B) > IP(Na-B) > OP(NB) > IP(NB) as evident from the decrease enthalpy values from Table 11. The entropy change is also more positive for OP(Na-B) > IP(Na-B) > OP(NB) > IP(NB) reflecting less orderliness at solid-solution interphase during adsorption.

**Table 11:** Thermodynamic parameters obtained for adsorption IP and OP by natural bentonite (NB) and activated sodium-bentonite (Na-B).

R	T(^o^K)	ΔG(KJ mol^-1^)	ΔS	ΔH	R^2^	ΔG	ΔS	ΔH	R^2^	ΔG	ΔS	ΔH	R^2^	ΔG	ΔS	ΔH	R^2^
		IP (Na-B)				OP (NA-B)				IP(NB)				OP(NB)			
8.314 (J mol^−1^ K^−1^)	283	− 61.43	0.102 (KJ mol^−1^ K^−1^)	− 32.33 (KJ mol^−1^)	0.82	− 65.29	0.108 (KJ mol^−1^ K^−1^)	− 34.66 (KJ mol^−1^)	0.79	−26.25	0.038(KJ mol^−1^ K^−1^)	− 15.33 (KJ mol^−1^)	0.79	− 33.98	0.048(KJ mol^−1^ K^−1^)	− 20.15 (KJ mol^−1^)	0.88
	293	− 62.46				− 66.37				− 26.64				−34.47			
	303	− 63.49				− 67.45				− 27.02				−34.96			
	313	− 64.52				− 68.54				− 27.41				−35.44			
	323	− 65.55				− 69.62				− 27.80				−35.93			
	333	− 66.58				− 70.70				− 28.18				−36.42			
	343	− 67.61				− 71.78				− 28.57				−36.91			
	353	− 68.63				− 72.87				− 28.95				−37.40			

### Recovery or reusability of spent bentonite

Wastewater treatment will be economical if the spent adsorbent can be recovered and regenerated. The bigger issue in wastewater treatment is the regeneration of the adsorbent materials. Regeneration is an alternative to disposal for spent both adsorbents^8,64^. The regeneration process is removing the pollutants from the spent natural and Na-bentonite, after cycles (3 trials) of repeated reuse of adsorbents for adsorption/desorption in the treatment. The recyclability of spent natural and Na-bentonite was evaluated under the same conditions cycles (3 trials) after recovery. It was found that the removal efficiency towards the investigated wastewater variables is less than the original utilized material by 5% and 4% in case of 1 M KCl and 3% and 2% in case of 5 μM Na_2_-EDTA for natural and Na-bentonite, respectively.

### Cost-of adsorbents

The adsorbent materials utilized in the present investigation are generally available at a relatively cheap rate, L.E. 500/ton for natural bentonite. The activated bentonite (Na) would cost approximately L.E. 850/ton for natural bentonite, sodium bicarbonate, including all expenses (utilities, transportation, power, handling, chemicals for recycling, accessories, electrical, and drying, etc.).

## Conclusion

The new method of bentonite activation, involving dehydration of clay at 300 °C, followed by rehydration with an aqueous solution of Na_2_CO_3_, enables easy preparation of Na- bentonite in two hours than present in the literature. The characterization and compositional analyses done by XRD, XRF, BET ESM, and FT-IR indicate the substitution of Ca^2+^ by Na^+^ in natural bentonite (NB). The results showed that the proportion of clay in sodium bentonite (Na-B) increased whereas the proportions of silt and sand decreased, which led to an increase in its surface area compared to natural bentonite (NB). Langmuir's isotherm model was better than the Dubinin-Radushkevich, Temkin, and Freundlich isotherm models for description equilibrium isotherm, while the pseudo-second-order model was the best fit for adsorption kinetic description. Modified or activated bentonite (Na-B) had a greater adsorption capacity when compared to natural bentonite (NB). Thermodynamically negative ΔG°, positive ΔH°, and positive ΔS° demonstrated that the adsorption processes are spontaneous and exothermic. The adsorbent Na-B used in this study exhibited a relatively good IP and OP adsorption capacity and removal yield compared to studies conducted with bentonite modified with different methods or with other adsorbents. In comparison to chemical-based approaches, it provides an environmentally acceptable a less expensive, safe, effective, and cost-effective or economic technique for wastewater treatment. The recyclability of spent natural and Na-bentonite demonstrated that the percentages of pollutant removal were sufficiently high.

Further study will be needed to verify the ability of the new Na-bentonite as an adsorbent to remove methyl violet color dye as well as examine this material's ability to adsorb contaminants simultaneously. The authors propose using Na-bentonite to wastewater treatment before discharge as well as expanding production of such material due to its high efficiency during the treatment process.

## Data Availability

All data generated or analyzed during this investigation are included in this published article.
